# Neuroprotective action of Cortexin, Cerebrolysin and Actovegin in acute or chronic brain ischemia in rats

**DOI:** 10.1371/journal.pone.0254493

**Published:** 2021-07-14

**Authors:** Denis V. Kurkin, Dmitry A. Bakulin, Evgeny I. Morkovin, Anna V. Kalatanova, Igor E. Makarenko, Artem R. Dorotenko, Nikolay S. Kovalev, Marina A. Dubrovina, Dmitry V. Verkholyak, Elizaveta E. Abrosimova, Alexey V. Smirnov, Maksim V. Shmidt, Ivan N. Tyurenkov

**Affiliations:** 1 Volgograd State Medical University (VSMU), Volgograd, Russia; 2 Pharm-Holding CJSC, St. Petersburg, Russia; Hungarian Academy of Sciences, HUNGARY

## Abstract

This study was the first to compare the neuroprotective activity of Cerebrolysin^®^, Actovegin^®^ and Cortexin^®^ in rodent models of acute and chronic brain ischemia. The neuroprotective action was evaluated in animals with acute (middle cerebral artery occlusion) or chronic (common carotid artery stenosis) brain ischemia models in male rats. Cortexin^®^ (1 or 3 mg/kg/day), Cerebrolysin^®^ (538 or 1614 mg/kg/day) and Actovegin^®^ (200 mg/kg/day) were administered for 10 days. To assess the neurological and motor impairments, open field test, adhesive removal test, rotarod performance test and Morris water maze test were performed. Brain damage was assessed macro- and microscopically, and antioxidant system activity was measured in brain homogenates. In separate experiments *in vitro* binding of Cortexin^®^ to a wide panel of receptors was assessed, and blood-brain barrier permeability of Cortexin^®^ was assessed in mice *in vivo*. Cortexin^®^ or Cerebrolysin^®^ and, to a lesser extent, Actovegin^®^ improved the recovery of neurological functions, reduced the severity of sensorimotor and cognitive impairments in rats. Cortexin^®^ reduced the size of necrosis of brain tissue in acute ischemia, improved functioning of the antioxidant system and prevented the development of severe neurodegenerative changes in chronic ischemia model. Radioactively labeled Cortexin^®^ crossed the blood-brain barrier in mice *in vivo* with concentrations equal to 6–8% of concentrations found in whole blood. During *in vitro* binding assay Cortexin^®^ (10 μg/ml) demonstrated high or moderate binding to AMPA-receptors (80.1%), kainate receptors (73.5%), mGluR1 (49.0%), GABAA1 (44.0%) and mGluR5 (39.7%), which means that effects observed *in vivo* could be related on the glutamatergic and GABAergic actions of Cortexin^®^. Thus, Cortexin, 1 or 3 mg/kg, or Cerebrolysin^®^, 538 or 1614 mg/kg, were effective in models acute and chronic brain ischemia in rats. Cortexin^®^ contains compounds acting on AMPA, kainate, mGluR1, GABAA1 and mGluR5 receptors *in vitro*, and readily crosses the blood-brain barrier in mice.

## 1 Introduction

Highly purified animal tissue extracts, containing tissue-specific peptides (cytomedins), are used in clinical practice in Russia and several post-Soviet republics. The preparations based on neurotrophic factors or blood components are used to correct the consequences of acute cerebrovascular events, including stroke and traumatic brain injury. The polypeptide complexes contained in the preparations Cerebrolysin^®^, Cortexin^®^, as well as more than 200 active components of hemodialysis product Actovegin^®^, demonstrated a multi-targeted neuroprotective effects in acute cerebral flow disorders, e.g. traumatic brain injury, vascular dementia and other neurological diseases [1–5].

Actovegin^®^ is a highly purified calf blood extract, containing more than 200 bioactive components with a molecular mass of <5 kDa, that improves brain metabolism and cognitive function in patients with cerebrovascular pathology [6].

Cortexin^®^ is obtained from the cerebral cortex of cattle and pigs, the use of which contributes to the survival and restoration of neurons, the improvement of memory formation processes, and restoration of brain functions in acute and chronic cerebrovascular disorders [2,7]. The chemical composition of Cortexin^®^ and Cerebrolysin^®^ seems to be similar because both preparations are obtained from brain tissues of cattle and pigs, but Cortexin^®^ could provide more selective action on the cortex cells, since it contains only the peptide fraction obtained from tissues of this brain structure [8], which determines the interest in studying the effects of this drug.

This study was the first performed to compare the neuroprotective activity of Cerebrolysin^®^, Actovegin^®^ and Cortexin^®^ in rodent models of acute and chronic brain ischemia.

## 2 Methods

To enhance the reproducibility of results presented in this study, a downloadable protocol file has been deposited at http://dx.doi.org/10.17504/protocols.io.brgam3se.

### 2.1 Ethics statement

All experiments were performed in accordance with the legislation of the Russian Federation and the technical standards of the Eurasian Economic Union for good laboratory practice (GOST R 53434–2009, GOST R 51000.4–2011) and Directive 2010/63/EU of the European Parliament and the Council of the European Union. The study protocol was reviewed and approved by the Regional Independent Ethics Committee (RNEK) Volgograd region, registration number: IRB 00005839 IORG 0004900 (OHRP), protocol No. 132 dated May 20, 2019.

### 2.2 Animals

The study used 224 male albino rats (300–350 g), purchased from laboratory animal house Stolbovaya (Moscow oblast, Russian Federation). After arrival the animals were quarantined for 14 days in the separate section of the vivarium. Throughout the experiment, rats were kept at 20 ± 2°C under conditions of 40–60% humidity and at stable day/night cycle (12/12 h) with unlimited access to food and water. The age of the animals at the beginning of the experiment was 40–42 weeks, which corresponded to the age of mature rats, and their body weight did not deviate from the average for all experimental groups by more than 20% (information on the weight of rats during the experiment as well as the main data of this study is available in a public repository (https://doi.org/10.6084/m9.figshare.13599776). Experiments performed in mice or *in vitro* are described in separate sections (see “Tissue distribution of Cortexin^®^” and “Receptor binding assay”). All painful manipulations were performed under general anesthesia with single intraperitoneal injection of zolazepam 20 mg/kg (Zoletil^®^100, Valdepharm, France) + xylazine 8 mg/kg (Xyla, Interchemie, Netherlands). Local anesthesia was performed with 2% lidocaine solution. When the experiment was completed, the animals were euthanized in a CO_2_-chamber.

### 2.3 Study design

**Fig 1:**
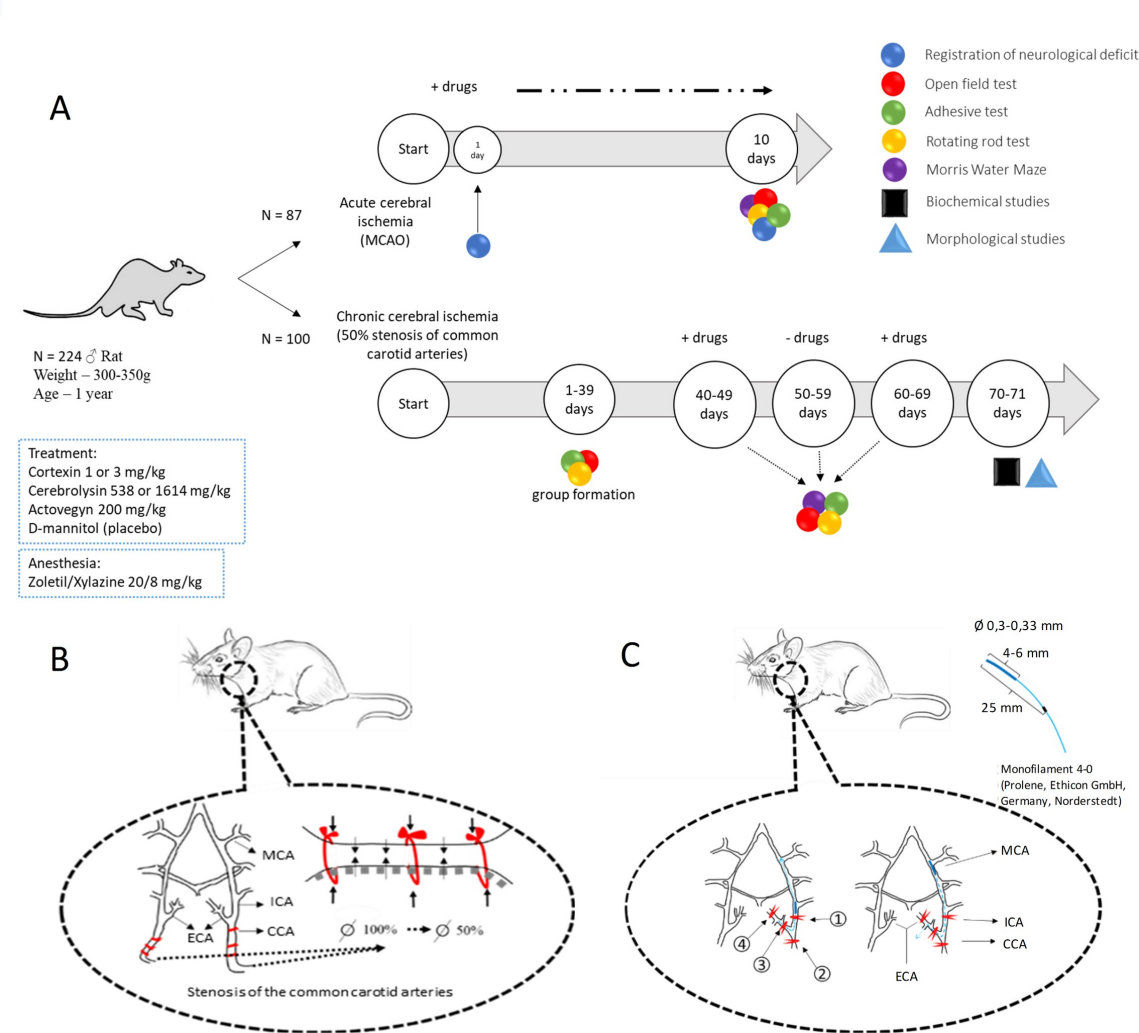
(A) Study design, (B) the principle of experimental modelling of chronic and (C) acute brain ischemia (Fig 1B Republished from Tyurenkov IN et al. (2020) [11] under a CC BY license, with permission from OOO “Media Sphere Publishing House”, original copyright 2020). CCA–common carotid artery; ECA–external carotid artery; ICA–internal carotid artery; MCA–middle cerebral artery; MCAO–middle cerebral artery occlusion.

The study design is shown on Fig 1A. In the first series of experiments, the studied drugs were administered to animals within 10 days after modeling of acute brain ischemia (10-day follow-up), their effectiveness was assessed by the dynamics of neurological deficit (Garcia and Combs & D’Alecy scales [9,10]), the severity of motor and sensory-motor impairments (open field test, adhesive test, rotating rod test), cognitive functions (Morris water maze). At the end of the experiment the animals with acute cerebral ischemia were sacrificed to access the brain necrosis volume by 2,3,5-triphenyltetrazolium chloride staining, described below. The neuroprotective effect of the drugs was also studied in rats with chronic cerebrovascular insufficiency during two ten-day courses of treatment with a break of 10 days. The number of rats in groups with acute and chronic cerebral ischemia is presented in S1 File.

Between the series (after interim analysis of the results of 10-day follow-up in acute brain ischemia model), an additional experiment was performed in limited number of animals (n = 37) randomly assigned to 4 groups (intact (n = 10), placebo (n = 9), 3 mg/kg Cortexin^®^ (n = 9), and 538 mg/kg Cerebrolysin^®^ (n = 9)) to assess the impact of both drugs on brain necrosis volume within 3-day follow up. Motor and sensory-motor impairments were also evaluated in open field test and adhesive test, respectively.

### 2.4 Experimental model of acute brain ischemia

The principle of reversible middle cerebral artery occlusion [12] is shown on Fig 1B. The left carotid artery was isolated from nerve bundle; the ligatures were placed under the artery. Then the cranial bifurcation, internal and external branches of the carotid artery were distinguished. The ligatures were placed under the common carotid artery (#1), under the internal carotid artery (#2) and under the external carotid artery (#3 and #4). The ligatures were reversibly tightened in the following order: #1, #2 and #4. Then on a puncture hole was made in the external carotid artery into which a nylon occluder (monofilament 4–0; Prolene, Ethicon GmbH, Norderstedt, Germany) was inserted so that its silicone-coated end passed through the bifurcation and ended up in the internal carotid artery. Then the ligature knot #2 was weakened and, guided by the mark (20 mm from the end covered with silicone), the occluder was carried further down the vessel to a distance of 20–23 mm, after which the ligature #4 was irreversibly bandaged, and #3 was tightened reversibly, firmly fixing the occluder. A sterile moisturizing gel was applied to the wound surface, preventing the drying of the operating surface. The eyes of the animal were closed throughout the operation; blepharogel was applied to them to prevent drying of the cornea and blindness of the animal. At the final stage of the operation (after 90 min of ischemia), the occluder was removed with sequential pulling of ligature #3, the remaining ligatures (#1 and # 2) were removed. After this, the wound was treated with a 0.05% chlorhexidine solution and sutured.

### 2.5 Experimental model of chronic brain ischemia

The principle of the model of 50% carotid stenosis is shown on Fig 1C. In anesthetized animals both common carotid arteries were isolated and the nylon threads were placed under the vessels. The threads were tightened to fully arrest the blood flow. Then the nylon threads were alternately removed until the blood flow through the vessel resumed by 50% of the initial values [13]. Blood flow in the common carotid arteries was recorded by the Doppler method. After this, the wound was treated with a 0.05% chlorhexidine solution and sutured.

### 2.6 Behavioral tests

#### 2.6.1 Neurological deficit

The neurological deficit was assessed with 9 point stroke-index scale, described by Combs & D’Alecy [9], and with 18 point scale, described by Garcia [10] in rats with acute or chronic brain ischemia. Combs & D’Alecy scale allows to reveal extrapyramidal disorders and include tests that evaluate muscle strength, tenacity, traction and balance of animals. A lower total score corresponded to more pronounced neurological impairment in rats. The Garcia scale determines the severity of the following indicators: muscle tone, motor activity, basic physiological reflexes, coordination of movement, sensitivity to touch.

#### 2.6.2 Sensorimotor functions

Sensorimotor functions were assessed in adhesive removal test. Briefly, on the palmar surface of upper extremities 2 squares of duct tape (5 mm^2^) were applicated, and the further behavior of the animal was assessed [14].

#### 2.6.3 Motor coordination

Motor coordination was assessed in rotating rod test. The latency to fall off the rotarod within 3 minutes (25 rpm) is recorded [15].

#### 2.6.4 Motor functions

Total distance passed was assessed in open field test within 3-min session [16].

#### 2.6.5 Cognitive impairment

**Fig 2:**
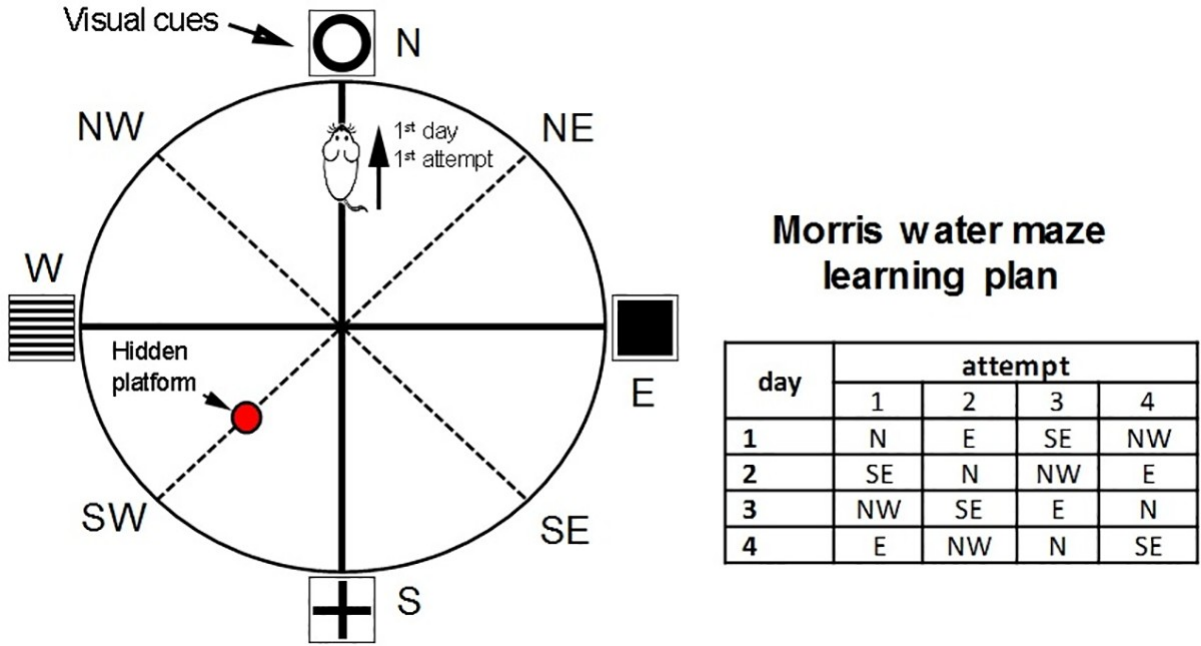
The schematic representation of Morris water maze apparatus and the order of animal testing.

Cognitive functions were assessed in Morris water maze test. When training for 4 training days, the animal was placed in water at a distance of 10 cm from the pool wall according to a certain pattern and the latent period of the flooded platform was recorded (fixed position; Fig 2). Each animal was placed in the apparatus 4 times for a maximum of 1 min with a break between sets of 30 s. If the rat did not find the platform for 1 min, it was placed on the platform and left for 15 s, after which the training was continued according to the above-indicated pattern [17].

### 2.7 Brain necrosis volume

Brain necrosis volume was assessed in 2 mm brain slices (6 per a brain) prepared with matrix BS-A–6000C (Braintree, USA) after 15 min of incubation with 1% 2,3,5-triphenyltetrazolium chloride at 37°С. After staining, the slices were photographed on a digital camera in the same plane with a millimeter ruler. The area of dyed and unpainted tissue was measured using ImageJ 1.37 software (NIH, Bethesda, MD, USA). The adjusted volume of the infarct zone was calculated as a percentage of the volume of the intact hemisphere [18]. Brain necrosis volume was assessed only in animals with acute brain ischemia after 3-day follow-up in additional experiment performed between the main series (see 2.3 Study design).

### 2.8 Antioxidant activity

Malonic dialdehyde (MDA) concentration in brain homogenates was assessed with thiobarbituric acid reaction [19]; the reduced glutathione levels were assessed in the reaction with 5,5-dinitrobis-(2-nitrobenzoic acid) [20]; catalase activity and the concentrations of lactate and pyruvate were assessed with commercially available reagents. Superoxide dismutase (SOD) was assessed with photometric method, based on inhibition of epinephrine oxidation [21]. All reactions were performed in triplicates. The antioxidant activity was assessed only in animals with chronic brain ischemia.

### 2.9 Morphometric analysis

Damage to brain tissue was evaluated by standard methods of morphometric studies. The degree of damage to neurons was evaluated according to the following method. The neurons were divided into three groups: normal or unchanged neurons; slightly modified neurons with preservation of the nucleus, but with structural or tinctorial disorders of the components of the cytoplasm (swelling, hyperchromatosis, chromatolysis, central tinctorial acidophilia); roughly altered neurons–pronounced wrinkling, “severe change”, homogenizing change in neurons, shadow cells. Morphometry was performed by staining the sections according to the Nissl method. The relative numerical density of unchanged neurons and neurons with mild and pronounced changes was determined. Microphotography of histological preparations was carried out with an Olympus digital camera (Japan) using a MICROS microscope (Austria). The morphometry was performed only in animals with chronic brain ischemia.

### 2.10 Tissue distribution of Cortexin^®^

In a separate experiment on male laboratory mice (n = 15 per group), the distribution of the Cortexin^®^ labeled with the radioactive isotope ^125^I was determined. The study drug was administered once or intravenously or intramuscularly to the animals at a dose of 3 or 30 mg/kg; after 0.5, 2 or 4 hours, animals (5 animals from each group) were killed by decapitation to determine the content of the radioactive label. Albumin (intravenously at a dose of 100 mg/kg) or Cerebrolysin^®^ (intramuscularly at a dose of 2.5 mg/kg), labeled with the radioactive isotope ^125^I; animals from the control group were injected with saline intravenously. Labeling of the studied drug and comparison drugs was carried out in the presence of chloramine T using sodium iodide containing the radioactive isotope ^125^I (9.25 GBq/ml). At each time point, the content of the radioactive label in whole blood, brain, heart, lungs, liver and spleen was determined by scintillometry method (calculated on the weight of the sample). Given the fact that Actovegin^®^ has different origin compared to other drugs and lacks specific brain-derived peptides, this drug was not used in this experiment.

### 2.11 Receptor binding assay

Cortexin^®^ binding was tested *in vitro* at 10.0 μg/mL in a wide panel of human and rodent receptors (See S2 File). Compound binding was calculated as a % inhibition of the binding of a radioactively labeled ligand specific for each target. Cellular agonist effect was calculated as a % of control response to a known reference agonist for each target and cellular antagonist effect was calculated as a % inhibition of control reference agonist response for each target.

### 2.12 Study drugs administration

The therapeutic dose of Cortexin^®^ was 1 mg/kg/day [22], and a dose 3 mg/kg/day was used as a triple therapeutic dose to assess the dose-dependency. For Cerebrolysin^®^ the respective doses were 538 and 1614 mg/kg/day [23]. Actovegin^®^ was used in a dose of 200 mg/kg/day only [24]. Drugs were injected intramuscularly.

### 2.13 Statistical analysis

Statistical analysis was performed with Microsoft Office Excel 2016 (Microsoft, USA) and Prism 6 (GraphPad Software Inc., USA). To the normality of the distribution was assessed with the Shapiro-Wilk test. Parametric data were compared using one-way analysis of variance (One-Way ANOVA) and t-student test with Bonferroni correction. Nonparametric data were compared using the Kruskal-Wallis rank analysis and the Dunn *post-hoc* test. For testing relationships between categorical variables (adhesive tape test performance) was used χ^2^ test. The differences were considered statistically significant at p <0.05. All data presented as the mean and standard error of the mean (SEM) or median and interquartile range [Q1;Q3] (for neurological tests).

## 3 Results

### 3.1 Neurological deficit

#### 3.1.1 Acute brain ischemia (10-day follow-up)

After occlusion of the left middle cerebral artery, the animals developed a pronounced neurological deficit according to the Garcia and Combs & D’Alecy scales: 3 hours after the operation the animals had similar symptoms of neurological deficit (circling, weakness of the limbs, impaired coordination, etc.; median Garcia score = 9.5 [8;10.3]; median Combs & D’Alecy score = 4 [3.8;4.3]) (Table 1). Within 10 days after left middle cerebral artery occlusion and course administration of the studied drugs, in all groups, the severity of neurological deficit according to the Garcia and Combs & D’Alecy scales decreased. In the groups receiving 1 or and 3 mg/kg/day Cortexin^®^, as well as 538 or 1614 mg/kg/day Cerebrolysin^®^, the median score of neurological deficit was significantly lower (p<0.05 for both scales), which indicates a pronounced neuroprotective effect of these drugs.

**Table 1:** The severity of neurological deficit according to the Garcia and Combs & D’Alecy scales, observed 3 and 24 hours after acute brain ischemia modeling, as well as after 10 days of treatment.

Group	n	Garcia			Combs & D’Alecy		
		3 h	24 h	10 days	3 h	24 h	10 days
**Intact**	15	18 [18;18]	18 [18;18]	18 [18;18]	9 [9;9]	9 [9;9]	9 [9;9]
**Placebo**	12	9.5 [8;10.3]#	13 [12.8;14]#	15.5 [15;16]#	4 [3.8;4.3]#	6 [6;6]#	7 [6;7]#
**Cortexin**^**®**^ **(1 mg/kg)**	12	9.5 [9;11]	14 [13.8;14]	18 [16;18]*	4 [3;4]	5.5 [4.8;6.3]	8 [8;9]*
**Cortexin**^**®**^ **(3 mg/kg)**	12	10 [9.8;11]	13.5 [12.8;14.5]	17.5 [17;18]*	4 [3;4]	5 [5;6]	8 [8;8.3]*
**Cerebrolysin**^**®**^ **(538 mg/kg)**	12	10 [9.8;11]	13 [12;14]	17 [17;18]*	4 [3;4]	5.5 [5;6]	9 [8;9]*
**Cerebrolysin**^**®**^ **(1614 mg/kg)**	12	9 [9;11]	13 [11;13.3]	18 [17;18]*	4 [3;4]	6 [5;7]	8.5 [7.8;9]*
**Actovegin**^**®**^ **(200 mg/kg)**	12	10.5 [9.8;11]	14 [13;14.3]	17 [16;18]	4 [3.8;4]	6 [5;6]	8 [7;9]

Note

#–p <0.05 compared to Intact

*–p <0.05 compared to placebo (Kruskal-Wallis rank analysis and the Dunn *post-hoc* test); data shown as median and interquartile range [Q1;Q3].

### 3.2 Sensomotor functions

#### 3.2.1 Acute brain ischemia (10-day follow-up)

**Fig 3:**
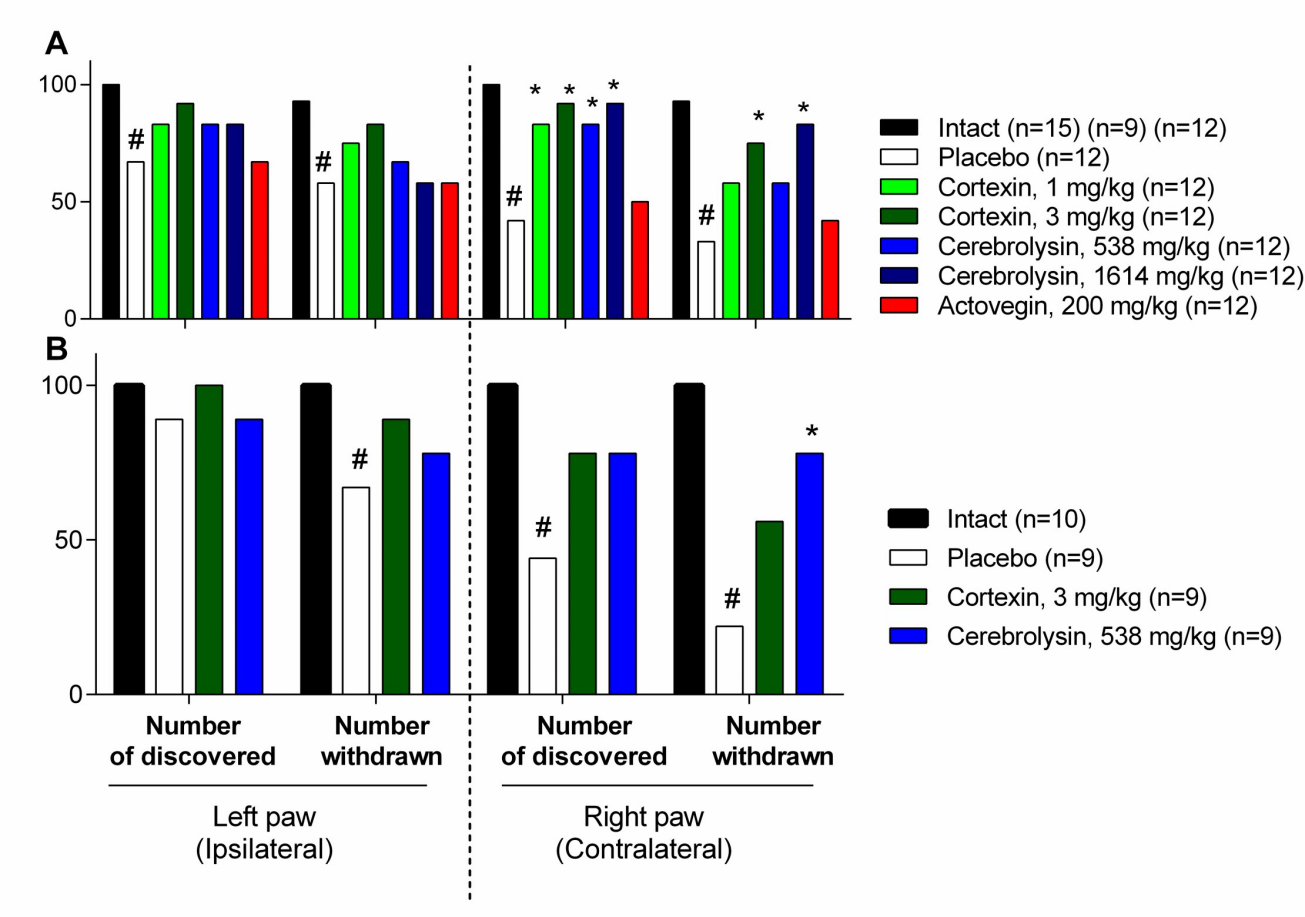
The number of animals that discovered and removed a foreign object during the adhesive tape test in 10- and 3-day follow-up experiments in acute brain ischemia model (A and B, correspondingly) (%). #–p <0.05 compared to Intact; *–p <0.05 compared to placebo group (χ^2^ test); data shown as the number of animals that discovered and removed the adhesive tape in the group (in %).

During adhesive test 5 of 12 animals from placebo group did not find the duct tape, and 4 of 12 found it, but subsequently did not try to remove it. In the groups receiving Cortexin^®^ at doses of 1 and 3 mg/kg/day or Cerebrolysin^®^ at doses of 538 and 1614 mg/kg/day, a significantly larger number of them detected a foreign object and removed it in less time. Actovegin^®^ showed inferiority to other drugs, and the animals in this group were indistinguishable from the animals in placebo group by the test results (Fig 3A).

#### 3.2.2 Acute brain ischemia (3-day follow-up)

In an additional experiment with 3-day follow-up, quite similar results were observed (Fig 3B). Interestingly, that even in placebo group the percentage of animals failed to find or remove the duct tape was almost equal to the values obtained in 10-day experiment, indicating that a decrease of performance in this test could be considered as a stable and obvious consequence of acute brain ischemia.

#### 3.2.3 Chronic brain ischemia

**Fig 4:**
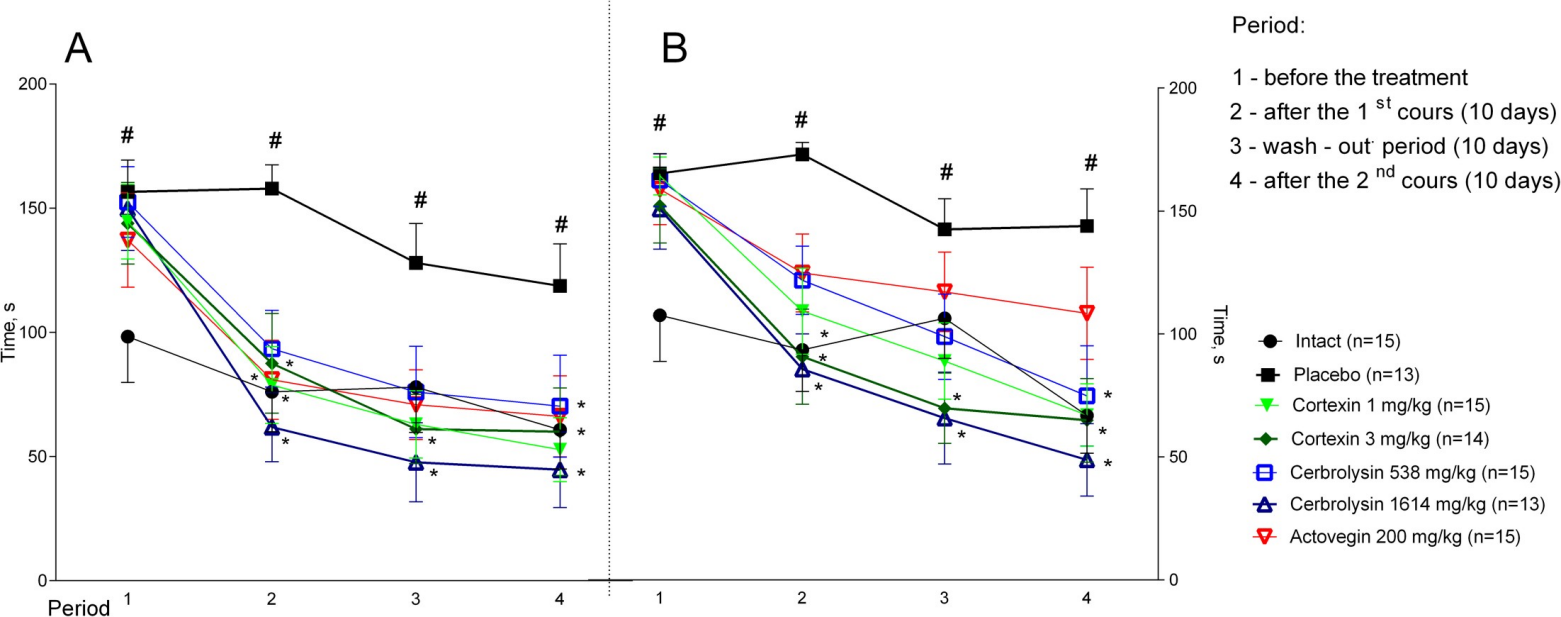
(A) The time discovered and (B) removed a foreign object during the adhesive tape test periods of experiments (after chronic brain ischemia) (s). #–p <0.05 compared to Intact; *–p <0.05 compared to placebo (Kruskal-Wallis rank analysis and the Dunn *post-hoc* test); data shown as the mean ± SEM.

The adhesive test in animals with chronic brain ischemia was performed 39 days after the surgery, the animals took more time to detect and remove the adhesive tape in comparison to intact animals. In groups receiving Cortexin^®^ at doses of 1 and 3 mg/kg or Cerebrolysin^®^ at a dose of 1614 mg/kg and Actovegin^®^, the latent period of detection and removal of the adhesive tape was significantly shorter than with those who were given a placebo (Fig 4).

### 3.3 Motor coordination

#### 3.3.1 Acute brain ischemia (10-day follow-up)

**Fig 5:**
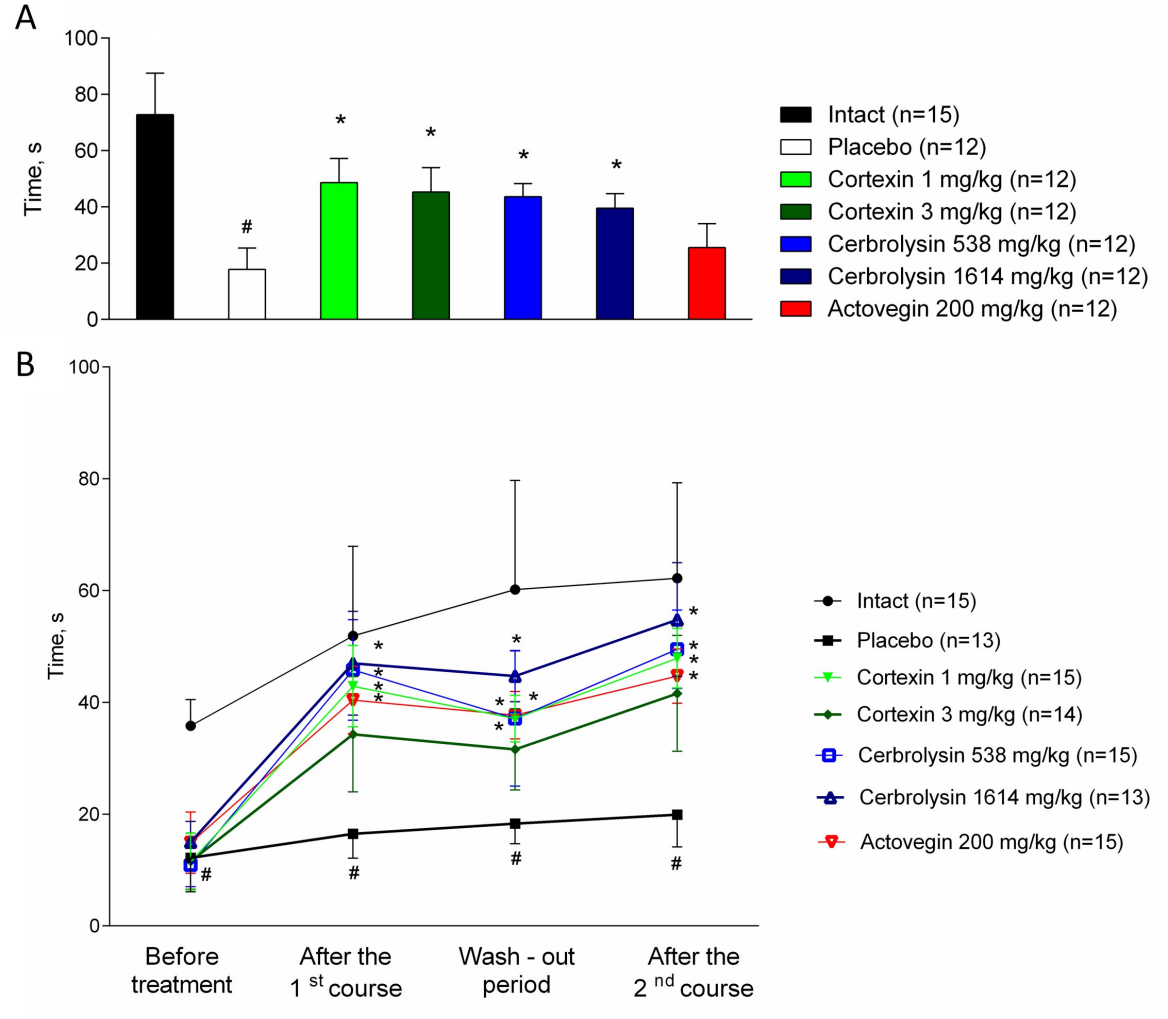
(A) Rotating rod performance in rats with acute and (B) chronic brain ischemia. #–p <0.05 compared to Intact; *–p < 0.05 compared to placebo (Kruskal-Wallis rank analysis and the Dunn *post-hoc* test); data shown as the mean ± SEM.

In the groups treated with Cortexin^®^ and Cerebrolysin^®^ in all doses, animals stayed much longer on a rotating rod, which indicates a higher strength and coordination during therapy (Fig 5A). In the group of animals that were administered Actovegin^®^, positive changes relative to the control were statistically insignificant.

#### 3.3.1 Chronic brain ischemia

When tested 39 days after surgery (before treatment), all operated animals had less time kept on a rotating rod in comparison to intact animals (Fig 5B). After 10 days of treatment, the performance of animals receiving placebo in this test did not change significantly. In the groups of animals with chronic brain ischemia treated with Cortexin^®^ at a dose of 1 mg/kg, Cerebrolysin^®^ at doses of 538 and 1641 mg/kg, as well as Actovegin^®^, the performance on a rotating rod was statistically better than in the group receiving a placebo, which indicates an increase in physical endurance and coordination of movements against the background of ongoing therapy with polypeptide drugs.

### 3.4 Motor functions

#### 3.4.1 Acute brain ischemia (10-day follow-up)

**Fig 6:**
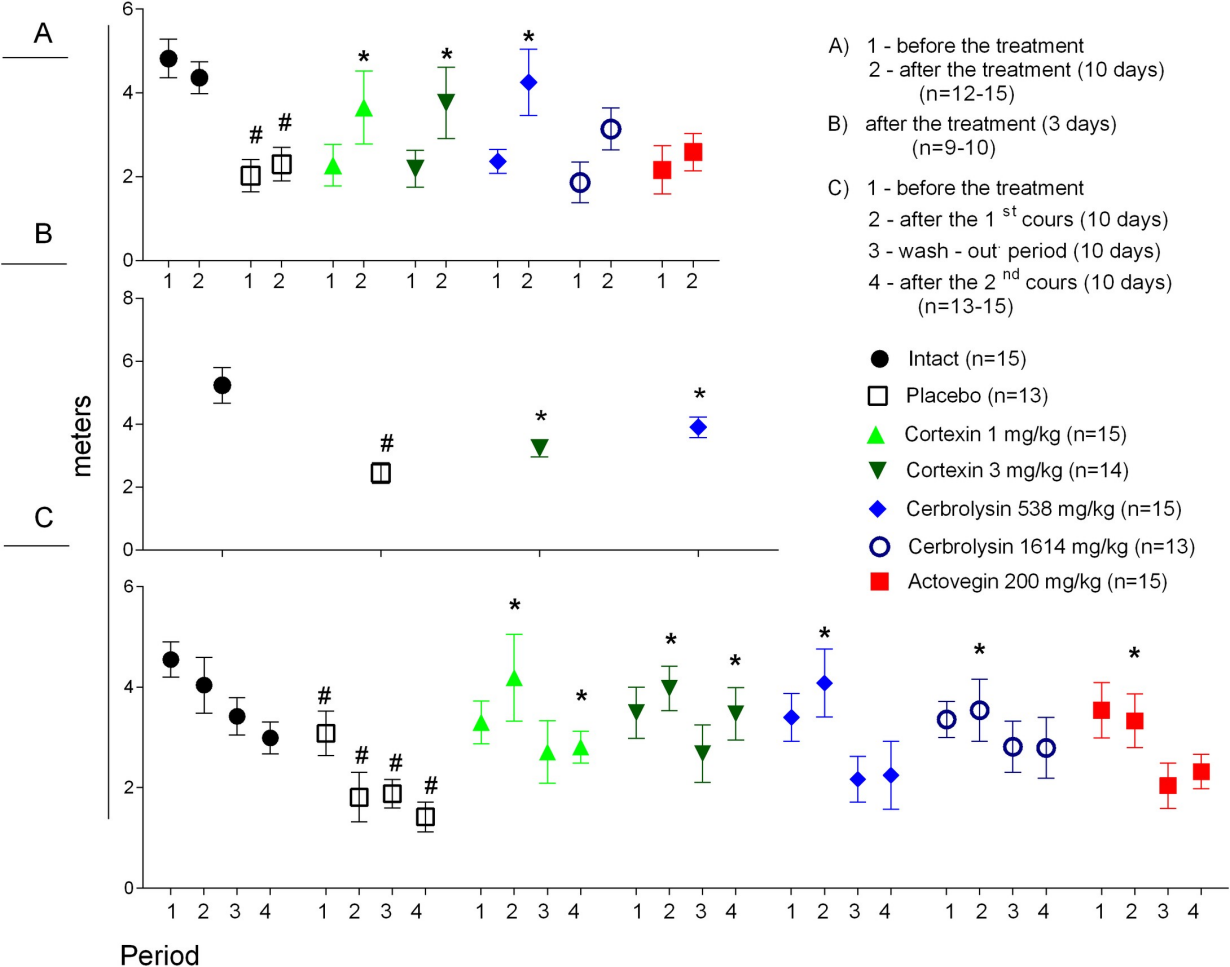
Motor activity in Open field test in rats with acute brain ischemia (after 10 (A) or 3 (B) days of treatment) and in rats with chronic brain ischemia (C). #–p <0.05 compared to Intact; *–p <0.05 compared to placebo (Kruskal-Wallis rank analysis and the Dunn *post-hoc* test); data shown as the mean ± SEM.

Movement disorders are common consequences of stroke, significantly reducing the quality of life of the patient and his relatives. Assessing the severity of psychomotor deficiency allows to determine the degree of damage to the brain and the effectiveness of pharmacotherapy. In the animals with left middle cerebral artery occlusion administered Сortexin^®^ or Сerebrolysin^®^ for 10 days (at doses of 1 or 3, or 538 mg/kg, respectively) a significant increase in locomotor activity was observed (Fig 6A).

#### 3.4.2 Acute brain ischemia (3-day follow-up)

In animals administered Cortexin^®^ at a dose of 3 mg/kg/day or Cerebrolysin^®^ at a dose of 538 mg/kg/day for 3 days, the motor activity was significantly higher compared to placebo group (p <0.05), and the total distance travelled was quite similar to the values obtained after 10-day follow-up experiment (Fig 6B).

#### 3.4.3 Chronic brain ischemia

Before the treatment all animals with chronic brain ischemia demonstrated lower motor activity compared to intact group. The courses of Cortexin^®^ (1 and 3 mg/kg) and Cerebrolysin^®^ (538 and 1641 mg/kg) significantly increased motor activity (Fig 6C). No consistent dynamics were observed during the repeated administration in chronic ischemia model (except for intact group), which could reflect the habituation of animals to the test conditions. However, in animals treated with study drugs the locomotor activity was higher than in placebo group.

### 3.5 Morris water maze

#### 3.5.1 Acute brain ischemia (10-day follow-up)

**Fig 7:**
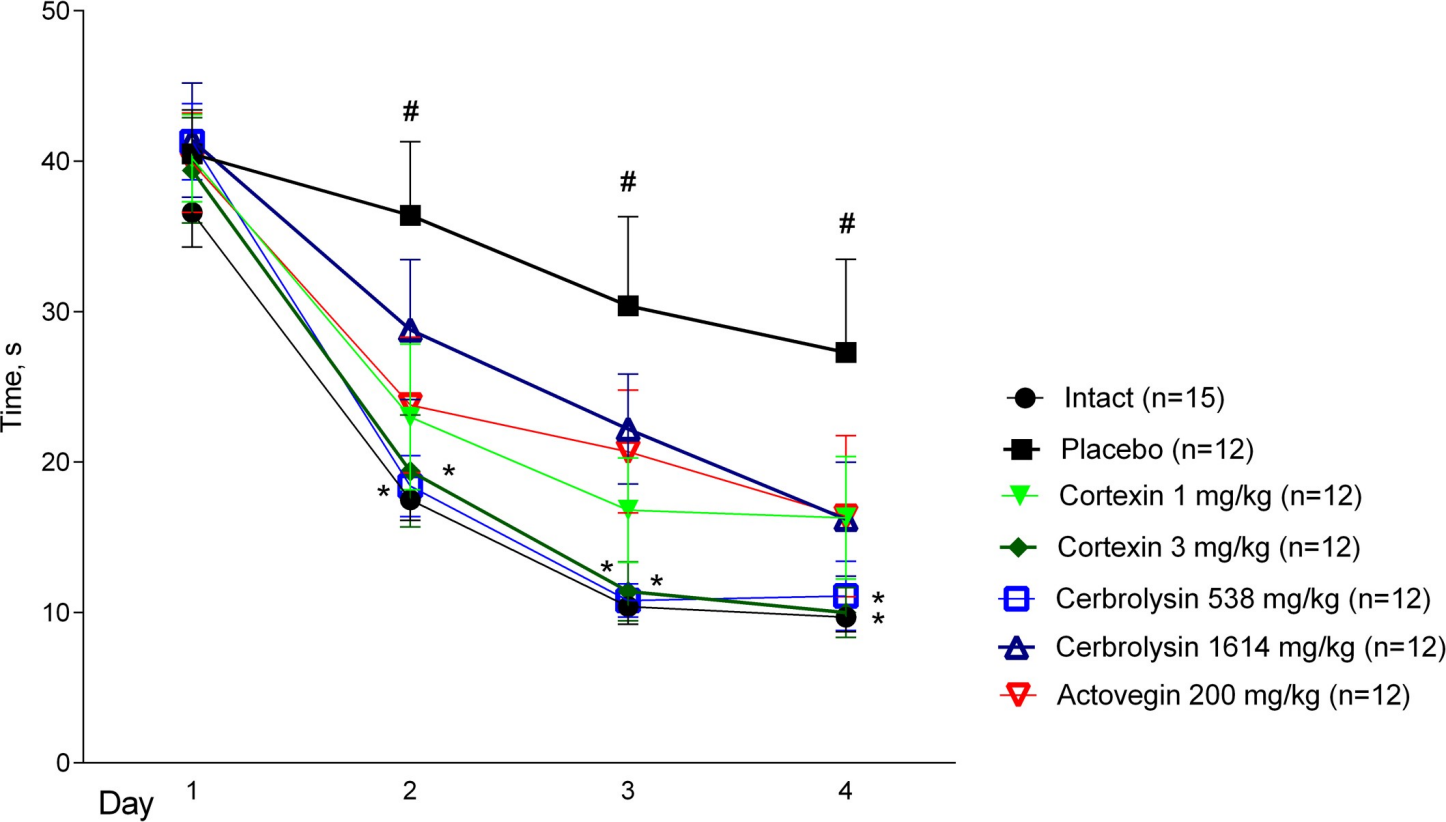
Morris water maze performance in rats during the course of therapy after left middle cerebral artery occlusion. #–p <0.05 compared to Intact; *–p <0.05 compared to placebo (Kruskal-Wallis rank analysis and the Dunn *post-hoc* test); data shown as the mean ± SEM.

From the 4^th^ day of treatment, the animals with acute brain ischemia were trained to find the platform in the Morris water maze, by the speed of which it is possible to judge the formation and preservation of spatial memory. On the 1^st^ day of training, when the animals first appeared in different parts of the pool, the average time spent on the platform for 4 attempts did not significantly differ between groups. On 2–4 days of training, the site was most quickly found by animals that were daily injected with Cortexin^®^ at a dose of 3 mg/kg or Cerebrolysin^®^ at a dose of 538 mg/kg (Fig 7).

#### 3.5.2 Chronic brain ischemia

In animals with chronic brain ischemia the Morris water maze test was carried out from 6 to 10 days after the start of treatment, and then 6 to 10 days after the treatment was canceled and from 6 to 10 days after the second course of therapy: the rat was placed in certain areas of the pool 4 times a day according to the same scheme and recorded the latent period of the location of the site (this data was partially shown earlier [11]).

**Fig 8:**
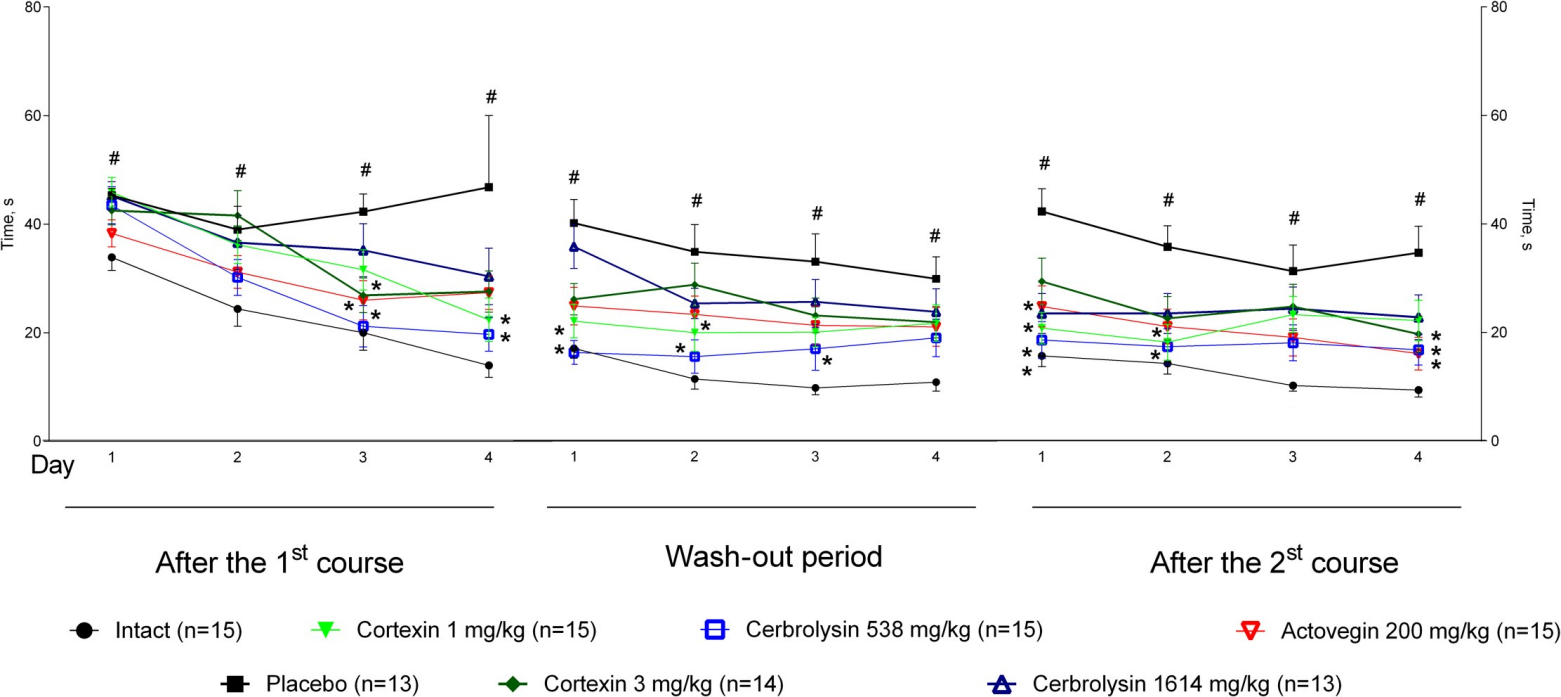
Morris water maze performance in rats with chronic brain ischemia. #–p <0.05 compared to Intact; *–p <0.05 compared to placebo (Kruskal-Wallis rank analysis and the Dunn *post-hoc* test); data shown as the mean ± SEM).

Throughout the experiment in the Morris water maze, animals with chronic brain ischemia given a placebo took more time to find a platform compared to intact group (p <0.05; Fig 8). At the end of the first treatment period on the fourth day of testing, the animals of the intact group and receiving Cortexin^®^ (1 or 3 mg/kg) (p <0.05), Cerebrolysin^®^ (538 or 1614 mg/kg) or Actovegin^®^, took less time to find a platform, than the animals from the placebo group, which may indicate better learning and preservation of spatial memory during treatment.

During the period of treatment cancellation, in the groups treated with Cortexin^®^ at doses of 1 and 3 mg/kg, as well as comparison drugs (Cerebrolysin^®^ at a dose of 538 mg/kg and Actovegin^®^) it was noted that, unlike the control ones, the animals remembered the location of the platform, since the time to search for it was significantly lower on the first day of testing. Moreover, in the group receiving Cortexin^®^, unlike Cerebrolysin^®^, the efficacy did not depend on the dose administered.

At the end of the repeated course of treatment, a significant improvement in cognitive functions relative to the control group was observed in animals that were injected with Cortexin^®^, Cerebrolysin^®^ or Actovegin^®^ in 1 TD or 3 TD. At the same time, interruptions in testing did not lead to the memory extinction, which was observed in animals receiving placebo. Thus, as a result of the study, a positive comparable effect of the course administration of Cortexin^®^ or Cerebrolysin^®^ on the formation and reproduction of Morris water maze performance was noted.

### 3.6 Antioxidant activity

#### 3.6.1 Chronic brain ischemia

**Fig 9:**
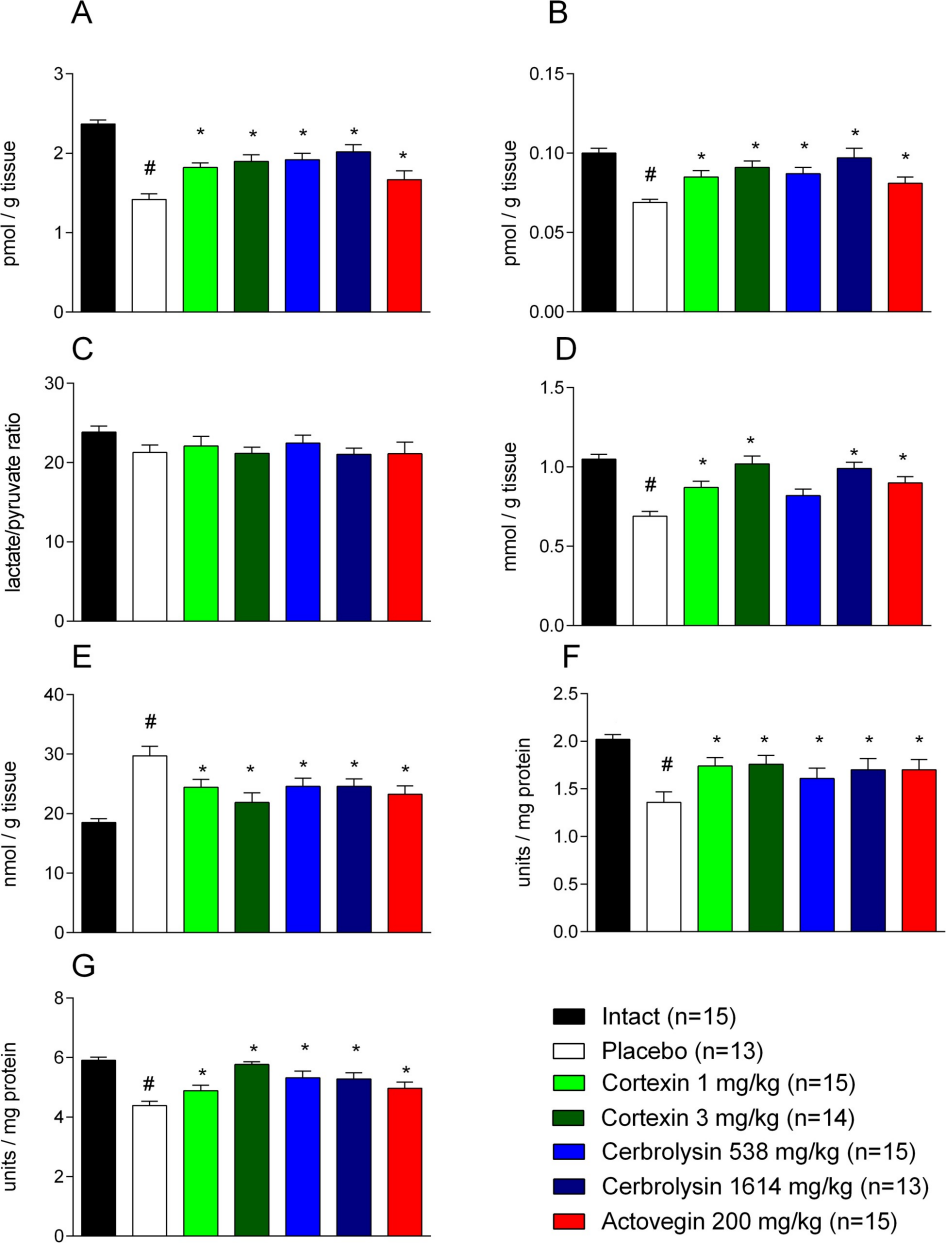
The activity of brain antioxidant systems in rats with chronic brain ischemia. (A) brain lactate levels; (B) brain pyruvate levels; (C) the relation of lactate to pyruvate in brain tissue; (D) brain glutathione levels; (E) malonic dialdehyde brain levels; (F) brain catalase activity; (G) brain superoxide dismutase activity; #–p <0.05 compared to Intact; *–p <0.05 compared to placebo (1-way ANOVA, t-student test with Bonferroni correction); data shown as the mean ± SEM.

In animals with chronic brain ischemia given a placebo, a statistically significant decrease in the content of lactate and pyruvate was recorded (p <0.001 when compared with the corresponding parameters recorded in rats from the control group), which was not accompanied by a significant decrease in their ratio (p> 0.05; Fig 9). In addition, experimental pathology led to the activation of free radical processes, as evidenced by a decrease in the reserves of reduced glutathione, the main antioxidant factor of cells and an increase in the concentration of MDA, the main marker of oxidative stress in rat brain homogenates, against the background of a decrease in the levels of SOD activity (superoxide is one of the main active forms of oxygen in the cell, and SOD plays a key role as an antioxidant) and catalase (p <0.001, in each case when compared with indicators in rats from the control group).

The effects of Cortexin^®^ and Cerebrolysin^®^ were dose dependent. In animals that were injected with Cortexin^®^, an increase in the content of lactate and pyruvate was observed (without a significant change in their ratio), an increase in the content of glutathione, restoration of SOD activity and, to a lesser extent, catalase, combined with a decrease in the concentration of MDA. The listed effects were most pronounced in rats to which Cortexin^®^ was administered at a dose of 3 mg/kg/day: an increase in SOD activity and a decrease in the concentration of MDA were most pronounced, and the average values of most indicators (except for the lactate content) did not statistically significantly differ from those of intact (p> 0.05 in each case), which indicates the ability of the drug Cortexin^®^ to activate antioxidant defense mechanisms in conditions of tissue hypoxia. In animals treated with Cerebrolysin^®^ at a dose of 1614 mg/kg/day, most of the indicators were comparable to those obtained in animals treated with Cortexin^®^ at a dose of 3 mg/kg/day (p <0.05). In animals treated with Actovegin^®^, the MDA content (p <0.05) was statistically significantly lower than in placebo group and was slightly higher than in control group.

### 3.7 Morphometry

#### 3.7.1 Acute brain ischemia (3-day follow-up)

**Fig 10:**
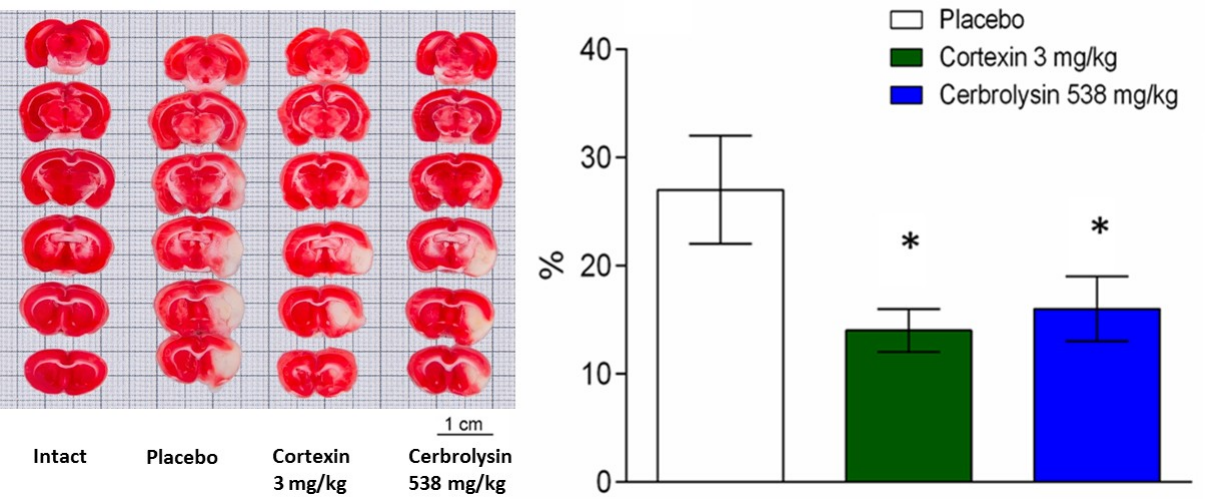
Representative photographs of brain slices obtained from animals with acute brain ischemia. Brain necrosis volume at 3 days (n = 9); *–p <0.05 compared to placebo (1-way ANOVA, t-student test with Bonferroni correction); data shown as the mean ± SEM.

When assessing the nature of changes in brain tissue in the left hemisphere, occurred by 3^rd^ day after acute brain ischemia modelling, a pronounced change in tissue color was noted in the sections from the blood supply zone of the middle cerebral artery–in the subcortical structures and on the periphery (Fig 10): in placebo group, in the cerebral cortex, in the area of the corpus callosum with adjacent subcortical white matter, striatum, the brain substance had pronounced changes, represented by homogeneous masses of white color without stratification of brain structures involving hippocampal fimbriae, corticofugal tracts, and the forebrain basal region. During morphometry, the average volume of necrosis in the placebo group was 26 ± 5% of the size of the left hemisphere. In the groups receiving Cortexin^®^ and Cerebrolysin^®^, the average size of the necrosis zone was smaller (by 45% and 38%, respectively).

#### 3.7.2 Chronic brain ischemia

**Fig 11:**
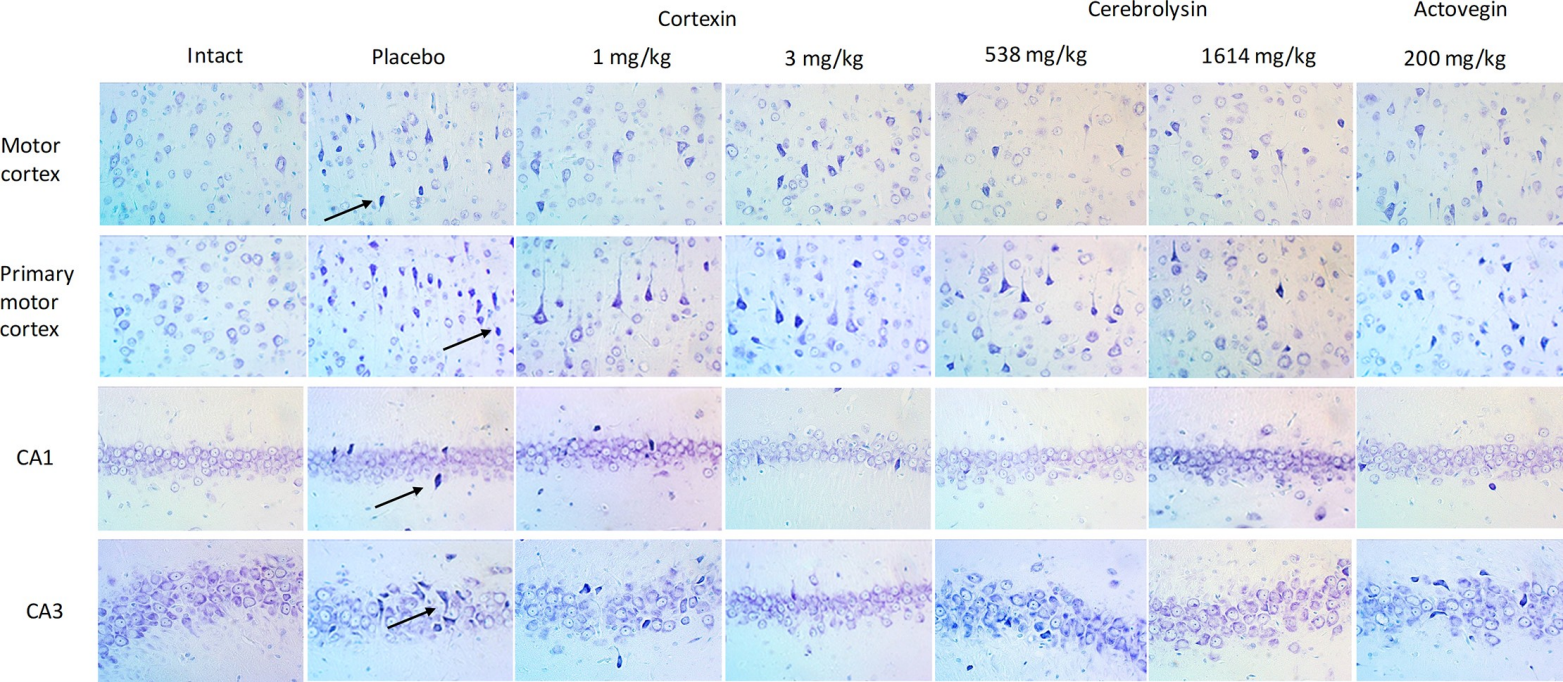
Representative microphotographs of hippocampal slices in animals with chronic brain ischemia. Arrows indicate the cells with roughly altered morphology, e.g. hyperchromia, triangular or polygonal form of perikaryon.

Modeling of chronic brain ischemia in rats via stenosis of the common carotid arteries caused mild and moderate pathomorphological changes in the neurons of the cerebral cortex (Fig 11). In the motor and somatosensory functional departments, pathomorphological changes, in most cases, were reduced to hyperchromia of the cytoplasm of the perikaryons and processes, a change in the shape of the perikaryons and an increase in the basophilia of the nuclei of the pyramidal layer neurons. In some neurons, signs of hydropic dystrophy with the appearance of single vacuoles in the cytoplasm of perikaryons were recorded. Pronounced pycnomorphic changes were noted only in individual cells. Compared with intact animals in the groups with chronic brain ischemia, the numerical density of damaged neurons was higher in the motor and, to a greater extent, somatosensory regions of the cerebral cortex.

Actovegin^®^ had a neuroprotective effect, manifested in a decrease in the degree of neurodegenerative changes in the somatosensory region of the cerebral cortex, but there was no significant effect in other parts of the brain. When studying the effectiveness of Cerebrolysin^®^, in the administered dose 538 mg/kg, it was noted that the numerical density of the damaged neurons in the somatosensory region of the cortex in these rats was statistically significantly lower compared to placebo. A comprehensive analysis of the morphological changes in the brain structures of rats with chronic brain ischemia during treatment with Cortexin^®^ revealed similar neuroprotective effect of the drug. The therapy of chronic cerebrovascular insufficiency with Cortexin^®^ at a dose of 1 and 3 mg/kg, this drug helped to reduce the number of damaged neurons in the somatosensory and motor areas of the cerebral cortex.

An analysis of the results of a morphological study allowed to conclude that the most significant protective effect on cerebral cortex structures was exerted by Cerebrolysin^®^ at a dose of 1614 mg/kg and Cortexin^®^ at doses of 1 and 3 mg/kg. The results of a pathomorphological study made it possible to explain the behavior of animals in psychopharmacological tests, in which typical and most obvious disorders of the central nervous system were fine motor skills.

**Fig 12:**
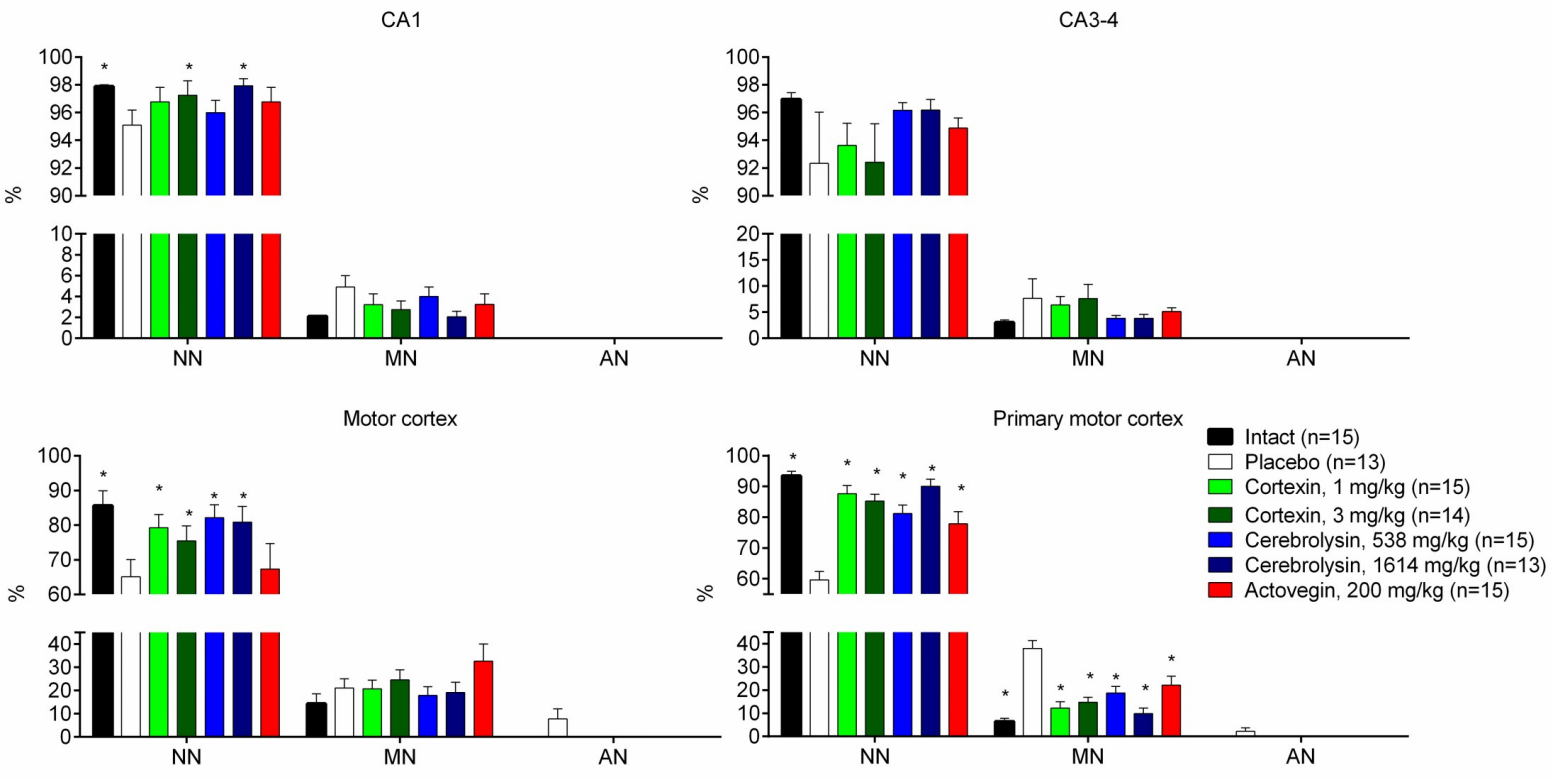
Hippocampal morphology in animals with chronic brain ischemia. NN–normal or unchanged neurons; MN–slightly modified neurons; AN–roughly altered neurons; #–p <0.05 compared to Intact; *–p <0.05 compared to placebo (Kruskal-Wallis rank analysis and the Dunn *post-hoc* test); data shown as the mean ± SEM.

The summarized data of the morphological studies are presented on Fig 12 and are arranged according to the degree of cell damage (this data was partially shown earlier [11]).

It is known that the hippocampus plays a significant role in the formation of memory, to a greater extent spatial, and therefore the hippocampus was taken to assess its morphological changes due to moderate cerebrovascular insufficiency. Morphological analysis of the structures of the hippocampus showed that 50% of the stenosis of the common carotid arteries led to weak or moderately expressed pathomorphological changes. In the fields CA1 and CA3 of the dorsal and ventral hippocampus, pathomorphological changes were characterized by weak pycnomorphic changes in single cells of pyramidal neurons. The numerical density of damaged neurons in animals with chronic brain ischemia and which were given a placebo was higher in both CA1 and CA3 fields of the hippocampus, however, significant differences from the group of intact animals were recorded only in CA1.

An analysis of morphological changes in the brain structures of rats with chronic cerebral ischemia and with the course of the drug Cortexin^®^ revealed an increase in the neuroprotective effect as the dose of the drug increases. Cortexin^®^ at a dose of 1 and 3 mg/kg reduced the number of damaged neurons in the somatosensory region of the cerebral cortex and contributed to the normalization of the histoarchitectonics of the CA1 field of the hippocampus. When studying brain tissue of animals that were injected with Cerebrolysin^®^ (1614 mg/kg), the numerical density of damaged neurons in the CA1 region of the hippocampus was the lowest, which indicates the protective effect of the drug.

According to the results of morphological data, we can conclude that the most significant neuroprotective effect was exerted by Cortexin^®^ in doses of 1 or 3 mg/kg and Cerebrolysin^®^ in a dose of 1614 mg/kg. The results of a pathomorphological study reflect the long-term consequences associated with the restriction of cerebral blood flow, allowing us to explain the behavior of animals in standard psychopharmacological tests, in which memory impairment was a typical and most obvious violation of the central nervous system. A cognitive impairment, including spatial memory disturbances, found in animals from the placebo group, and better indicators of cognitive function in animals treated with Cortexin^®^, Cerebrolysin^®^ and, to a lesser extent, with Actovegin^®^, are obviously the result of smaller structural changes in the brain tissue.

### 3.8 Cortexin^®^ tissue distribution

**Fig 13:**
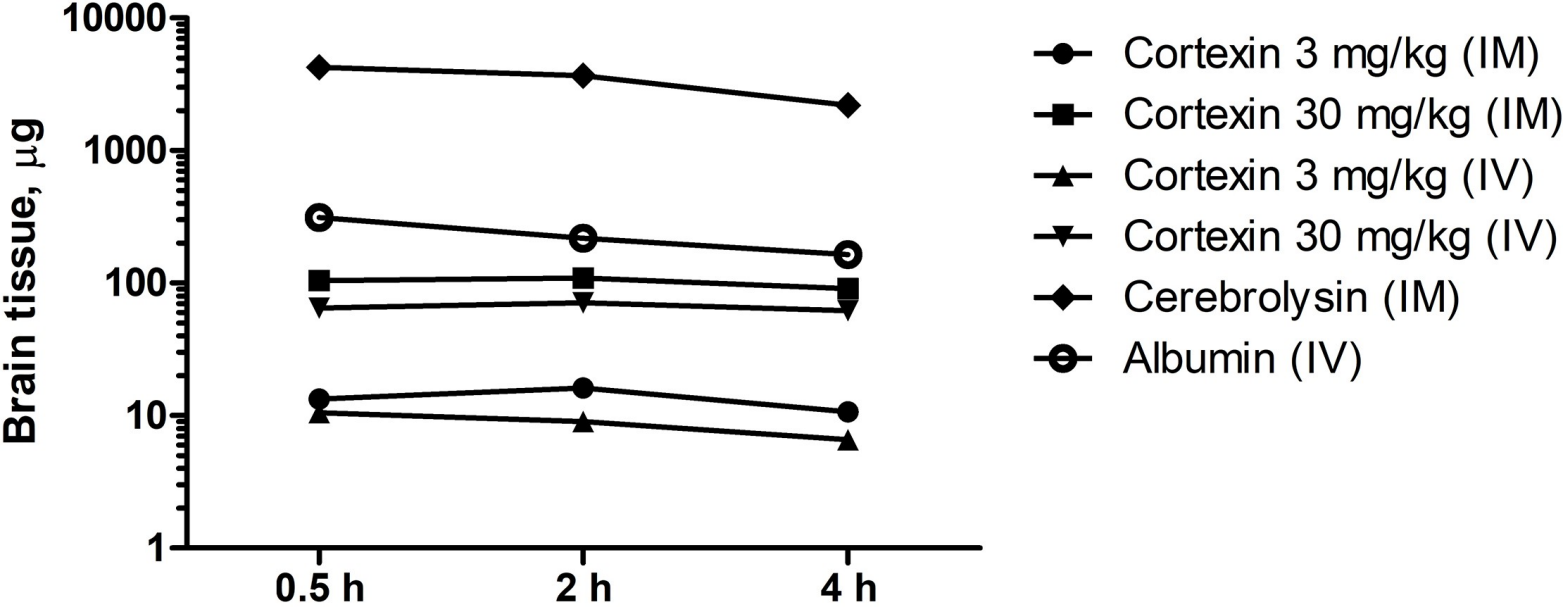
Brain content of ^125^I-labeled Cortexin^®^, Cerebrolysin^®^ and albumin in mice. Data shown as geometric mean (n = 15).

According to the results of a study of the distribution of radioactively labeled Cortexin^®^, already 30 min after the administration of the drug, regardless of the route of administration, the level of radioactivity in the brain tissues was 6–8% of that in whole blood (Fig 13), which indicates the ability of the drug to cross the blood-brain barrier of healthy animals. This indicator was higher than that of Cerebrolysin^®^ (about 5%) or albumin (about 3%).

### 3.9 Cortexin^®^ receptor binding

**Fig 14:**
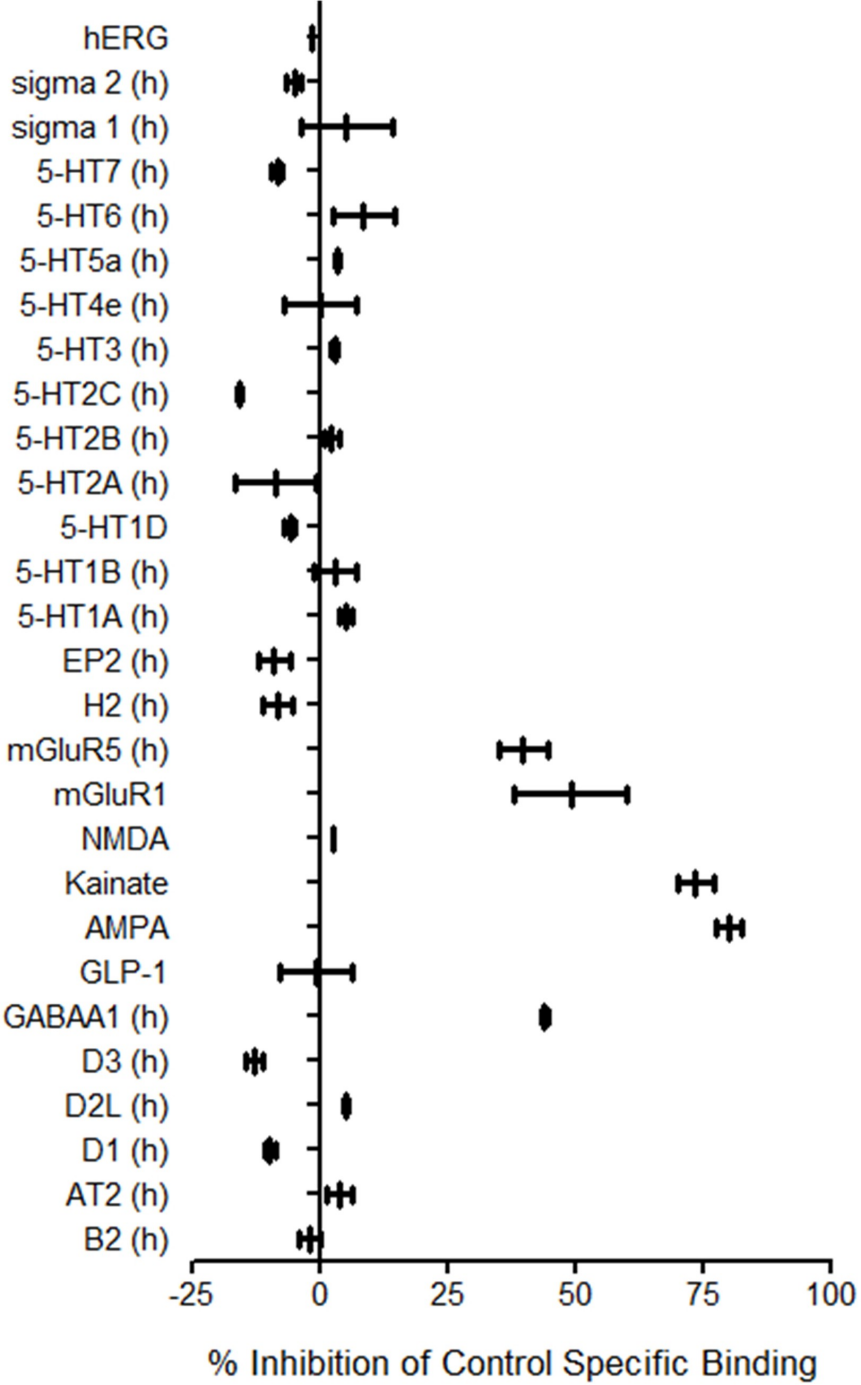
Cortexin^®^ receptor binding *in vitro*. Data shown as the median and range (n = 2).

Main results of receptor binding studies performed with Cortexin^®^ are shown at Fig 14. At 10 μg/ml Cortexin^®^ shown higher binding to AMPA-receptors (80.1%), kainate receptors (73.5%), mGluR1 (49.0%), GABAA1 (44.0%) and mGluR5 (39.7%). Thus, the effects observed *in vivo* could be related on the glutamatergic and GABAergic actions of Cortexin^®^.

## 4 Discussion

Stroke is the most common cause of sensory, motor, and coordinative disabilities. Pharmacological interventions in the acute management of stroke are aimed to improve cerebral circulation and to limit brain damage and poststroke complications [25]. However neuroprotective drugs which could be beneficial in such diseases still have a limited base of evidence. This study was planned to provide a direct comparison of neuroprotective effects of highly purified animal tissue extracts used in clinical practice in rat models of brain ischemia revealed that Cortexin^®^ or Cerebrolysin^®^ and, to a lesser extent, Actovegin^®^ improve the recovery of neurological functions, reduce the severity of sensorimotor and cognitive impairments.

With the accumulation of knowledge in the field of physiology and pathophysiology, as well as the development of biotechnology, it became possible to adjust the balance of factors ensuring the flow of normal metabolism, growth (growth factors) and cell development (factors affecting differentiation), which is especially important in extreme conditions (oxidative stress, ischemia, aging). An example of the isolated use of certain factors in experimental studies is the Klotho, GDF11, BDNF etc proteins, which over the past few years have been actively studied as candidates for drugs for treating some age-related diseases and prolonging active longevity [26–29].

Several neuropeptide preparations, including Cortexin^®^ and Cerebrolysin^®^, could mimic the effects of endogenous neurotrophic factors protecting the brain against the ischemic cascade and improving the neuroplasticity. Cerebrolysin^®^ is one of the most studied representatives of organ preparations; a large number of scientific papers have been published on its effectiveness in the treatment of neurological disorders. The dry residue of Cerebrolysin^®^ contains 15% protein and 85% amino acids. The role of amino acids can be to stabilize the spatial structure of proteins and to more evenly distribute them in solution due to non-specific interactions with the peptides of the drug. Also, the protective role of amino acids, the molecular mechanisms of which are listed in the table, cannot be ruled out. Improving the qualitative and quantitative characteristics of Cerebrolysin^®^ may lead to the development of a new drug with a better pharmacodynamic profile. The study of polypeptide preparations in modern conditions may set a new vector for scientific research in the field of detection and therapeutic use of various substances that can be obtained from animal organs. Cortexin^®^ is produced by the pharmaceutical company Geropharm by extraction from the cerebral cortex of cattle or pigs under 12 months of age. This drug is a complex of balanced neuropeptides (levorotatory amino acids). According to the results of a study of the composition and basic physicochemical properties of the Cortexin^®^ preparation, it was found that the preparation contains predominantly (from 70 to 95%) acidic and neutral polypeptides with a molecular weight of 1000 to 10,000 Da and an isoelectric point pI of 3.5–9.5. Thus, Cortexin^®^ and Cerebrolysin^®^ are similar in the production method, but significantly differ in their qualitative and quantitative composition [30,31].

In accordance to *in vivo* results, radioactively labelled Cortexin^®^ and Cerebrolysin^®^ crossed the blood-brain barrier in mice, thus the observed results could be attributed to the brain-specific actions of these preparations. The results obtained in this study showed that the drugs Cortexin^®^, 1 or 3 mg/kg, or Cerebrolysin^®^, 538 or 1614 mg/kg, were effective in models acute and chronic brain ischemia in rats, while Actovegin^®^ was inferior to the other drugs. Thus, we can conclude that specific neuropeptides, which are much more in the Cortexin^®^ preparation, play an important role in the implementation of the neuroprotective effect in case of cerebrovascular event.

The *in vitro* results show that Cortexin^®^ could affect the glutamate and GABAergic cascades with higher affinity to AMPA-receptors, kainate receptors, mGluR1, GABAA1 and mGluR5. Thus, the effects observed *in vivo* could be related on the glutamatergic and GABAergic actions of Cortexin^®^. In comparison to Cerebrolysin^®^, Cortexin^®^ could contain more neuron-specific proteins and fewer amino acids. Cerebrolysin^®^ had a similar pronounced and complex neuroprotective effects, but the dose at which this drug was administered was hundreds of times higher than the dose at which Cortexin^®^ was administered, which proves the high importance of the peptide fraction in the effectiveness of neuroprotective therapy.

The presented results show, that the development of polypeptide drugs for the treatment of the consequences of cerebrovascular accident is a relevant and promising direction. Probably further studies of polypeptide drugs will optimize their composition to improve the pharmacodynamic profile. As for other nonclinical studies, the results of this study should be interpreted with caution due to intrinsic limitations, including use of experimental pathology without any concomitant diseases (e.g. hypertension, obesity, dyslipidemia etc.) which a common in patients with stroke. The major limitation of the study is the use of mature animals (40–42 weeks of age), thus, an influence of aging, which is a single, non-modifiable risk factor for stroke [32–36], was not assessed in the acute brain ischemia model. At the end of chronic experiment, the age of the animals was about 1 year (at least one half-lifetime of rats), which also seems to be insufficient for extrapolation to patients aged > 65 years, demonstrating the highest rate of ischemic stroke occurrence. As the burden of ischemic stroke is higher in men [37], the use of male rats could be considered acceptable. Another limitation is that brain necrosis volume in acute 3-day experiment was assessed only in limited number of animals, so no groups were created for alternative dose levels of Cortexin^®^ and Cerebrolysin^®^, and for Actovegin^®^ administration. The method used for brain staining was found to be inappropriate for a 10-day experiment due to relatively fast replacement of necrotic tissue with connective tissue, which also positively reacted to 2,3,5-triphenyltetrazolium chloride due to presence of hydrogenases. Thus, we made a collective decision to maximally reduce the number of animals in additional experiment and to reduce the group number to 3, which led to lack of data on acute effects of Actovegin^®^ or other dose levels of Cortexin^®^ and Cerebrolysin^®^ on brain tissue. However, this is partially compensated with behavioral data collected in either 3-day or 10-day experiment. The last limitation of the study is that the effects of study medications were assessed only in ischemia models, while no data on their efficacy on the cerebral hemorrhages was collected. This needs further investigations, which are beyond the scope of the current work.

## 5 Conclusions

Cortexin^®^ (1 or 3 mg/kg) as well as Cerebrolysin^®^ (538 or 1614 mg/kg) were comparable effective in models acute and chronic brain ischemia in rats. Cortexin^®^ contains compounds acting on AMPA, kainate, mGluR1, GABAA1 and mGluR5 receptors *in vitro*, and readily crosses the blood-brain barrier in mice.

## Supporting information

S1 File Number of rats per group. (DOCX)

S2 File In vitro binding assays. (DOCX)
