# Supplementary material for: Neuroprotective action of Cortexin, Cerebrolysin and Actovegin in acute or chronic brain ischemia in rats
Source: PLoS One. 2021 Jul 14;16(7):e0254493. doi: 10.1371/journal.pone.0254493 (PMC8279368; doi:10.1371/journal.pone.0254493)
Supplement: S1 File — (DOCX) [file pone.0254493.s001.docx]

Table A. Number of rats per group.

| # | Group/Treatment | Number of rats in groups (n) | | |
| --- | --- | --- | --- | --- |
|  |  | Acute brain ischemia, treatment: | | Chronic brain ischemia |
|  |  | 3 days | 10 days |  |
| 1 | Intact (no treatment) | 10 | 15 | 15 |
| 2 | Placebo (D-mannitol) | 9 | 12 | 13 |
| 3 | Cortexin 1 mg/kg | - | 12 | 15 |
| 4 | Cortexin 3 mg/kg | 9 | 12 | 14 |
| 5 | Cerebrolysin 538 mg/kg | 9 | 12 | 15 |
| 6 | Cerebrolysin 1614 mg/kg | - | 12 | 13 |
| 7 | Actovegyn 200 mg/kg | - | 12 | 15 |
