# Supplementary material for: Neuroprotective action of Cortexin, Cerebrolysin and Actovegin in acute or chronic brain ischemia in rats
Source: PLoS One. 2021 Jul 14;16(7):e0254493. doi: 10.1371/journal.pone.0254493 (PMC8279368; doi:10.1371/journal.pone.0254493)
Supplement: S2 File — (DOCX) [file pone.0254493.s002.docx]

Table B. In vitro binding assays.

| **Assay** | **Source** | **Ligand** | **Concentration** | **K_d_** | **Non specific** | **Incubation** |
| --- | --- | --- | --- | --- | --- | --- |
| β2 (h) (antagonist radioligand) | Human recombinant (CHO cells) | [3H](-)CGP 12177 | 0.3 nM | 0.15 nM | Alprenolol (50 μM) | 120 min RT |
| AT2 (h) (agonist radioligand) | Human recombinant (HEK-293 cells) | [125I]CGP 42112A | 0.01 nM | 0.01 nM | angiotensin-II (1 μM) | 4 hr 37°C |
| D1 (h) (antagonist radioligand) | Human recombinant (CHO cells) | [3H]SCH 23390 | 0.3 nM | 0.2 nM | SCH 23390 (1 μM) | 60 min RT |
| D2L (h) (antagonist radioligand) | human recombinant (HEK-293 cells) | [3H]methylspiperone | 0.3 nM | 0.1 nM | butaclamol (10 μM) | 60 min RT |
| D3 (h) (antagonist radioligand) | Human recombinant (CHO cells) | [3H]methylspiperone | 0.3 nM | 0.085 nM | (+)butaclamol (10 μM) | 60 min RT |
| GABAA1 (h) (α1,β2,γ2) (agonist radioligand) | human recombinant (CHO cells) | [3H]muscimol | 15 nM | 30 nM | muscimol (10 μM) | 120 min RT |
| GLP-1 (agonist radioligand) | mouse endogenous (beta TC6 cells) | [125I]GLP-1(7-36) | 0.025 nM | 0.1 nM | GLP-1(7-36) (1 μM) | 120 min 37°C |
| mGluR1 (agonist radioligand) | rat cerebellum | [3H]quisqualate | 40 nM | 240 nM | L-Glutamate (1 mM) | 60 min RT |
| mGluR5 (h) (agonist radioligand) | human recombinant (CHO cells) | [3H]Quisqualate | 40nM | 44nM | L-Glutamate (1mM) | 120 min RT |
| H2 (h) (antagonist radioligand) | human recombinant (CHO cells) | [125I]APT | 0.075 nM | 2.9 nM | tiotidine (100 μM) | 120 min RT |
| EP2 (h) (agonist radioligand) | human recombinant (HEK-293 cells) | [3H]PGE2 | 3 nM | 3 nM | PGE2 (10 μM) | 120 min RT |
| 5-HT1A (h) (agonist radioligand) | human recombinant (HEK-293 cells) | [3H]8-OHDPAT | 0.5 nM | 0.5 nM | 8-OH-DPAT (10 μM) | 60 min RT |
| 5-HT1B (h) (antagonist radioligand) | human recombinant (Chem-1 (RBL) cells) | [3H]GR125743 | 1 nM | 0.8 nM | Serotonine (10μM) | 60 min 37°C |
| 5-HT1D (agonist radioligand) | rat recombinant (CHO cells) | [3H]serotonin | 1 nM | 0.5 nM | serotonin (10 μM) | 60 min RT |
| 5-HT2A (h) (agonist radioligand) | human recombinant (HEK-293 cells) | [125I](±)DOI | 0.1 nM | 0.3 nM | (±)DOI (1 μM) | 60 min RT |
| 5-HT2B (h) (antagonist radioligand) | human recombinant (CHO cells) | [3H]mesulergine | 2 nM | 2.4 nM | SB206553 (10 μM) | 60 min RT |
| 5-HT2C (h) (agonist radioligand) | human recombinant (HEK-293 cells) | [125I](±)DOI | 0.1 nM | 0.9 nM | (±)DOI (10 μM) | 60 min 37°C |
| 5-HT4 e (h) (antagonist radioligand) | human recombinant (CHO cells) | [3H]GR 113808 | 0.3 nM | 0.15 nM | serotonin (100 μM) | 60 min 37°C |
| 5-HT5a (h) (agonist radioligand) | human recombinant (HEK-293 cells) | [3H]LSD | 1.5 nM | 1.5 nM | serotonin (100 μM) | 120 min 37°C |
| 5-HT6 (h) (agonist radioligand) | human recombinant (CHO cells) | [3H]LSD | 2 nM | 1.8 nM | serotonin (100 μM) | 120 min 37°C |
| 5-HT7 (h) (agonist radioligand) | human recombinant (CHO cells) | [3H]LSD | 4 nM | 2.3 nM | serotonin (10 μM) | 120 min RT |
| sigma 1 (h) (agonist radioligand) | human endogenous (Jurkat cells) | [3H] (+)pentazocine | 15 nM | 16 nM | haloperidol (10 μM) | 120 min 37°C |
| sigma 2 (h) (agonist radioligand) | human endogenous (Jurkat cells) | [3H] DTG (+1μM (+) Pentazocine) | 25 nM | 80.84 nM | Haloperidol (10 μM) | 60 min RT |
| AMPA (agonist radioligand) | rat cerebral cortex | [3H]AMPA | 8 nM | 82 nM | L-glutamate (1 mM) | 60 min 4°C |
| Kainate (agonist radioligand) | rat cerebral cortex | [3H]kainic acid | 5 nM | 19 nM | L-glutamate (1 mM) | 60 min 4°C |
| NMDA (antagonist radioligand) | rat cerebral cortex | [3H]CGP39653 | 5 nM | 23 nM | L-glutamate (100 μM) | 60 min 4°C |
| 5-HT3 (h) (antagonist radioligand) | Human recombinant (CHO cells) | [3H]BRL 43694 | 0.5 nM | 1.15 nM | MDL 72222 (10 μM) | 120 min RT |
| Potassium Channel hERG (human) | Human recombinant (HEK-293 cells) | [3H]Dofetilide | 3 nM | 6.6 nM | Terfenadine (25 μM) | 60 min RT |
